# A double‐lumen catheter for hemodialysis dislocated into the mediastinum

**DOI:** 10.1002/ccr3.2326

**Published:** 2019-08-04

**Authors:** Takuya Kusumoto, Kento Mitsushio, Nobuyuki Kajiwara

**Affiliations:** ^1^ Post Graduate Clinical Education Center Ikeda City Hospital Ikeda Japan; ^2^ Department of Endocrinology and Metabolism Ikeda City Hospital Ikeda Japan; ^3^ Department of Nephrology Ikeda City Hospital Ikeda Japan

**Keywords:** complication, hemodialysis catheter, mediastinum, vascular access devices

## Abstract

The insertion of a catheter into the mediastinum can occur in any patient as a complication. We must check for blood regurgitation not only in the blood removal line but also in the blood return line.

A 71‐year‐old woman with chronic renal failure presented with uremia, and she did not have an internal shunt. Therefore, we inserted a double‐lumen catheter for hemodialysis through the right internal jugular vein. We needed to rotate the guidewire due to some resistance. The guidewire in the vein could be visualized by ultrasonography. However, the blood removal line showed no blood regurgitation. After we pulled the catheter out by 2 cm, we observed blood regurgitation. A chest X‐ray revealed an appropriately positioned catheter (Figure 1). However, we could not obtain blood flow for hemodialysis. Then, we injected a contrast agent into the blood return line. Chest X‐ray and CT revealed contrast agent leakage in the mediastinum (Figures 2 and 3). We removed the catheter without any complications of catheter insertion and contrast agent injection. After 4 days, the contrast agent disappeared on the chest X‐ray (Figure 4).

**Figure 1:**
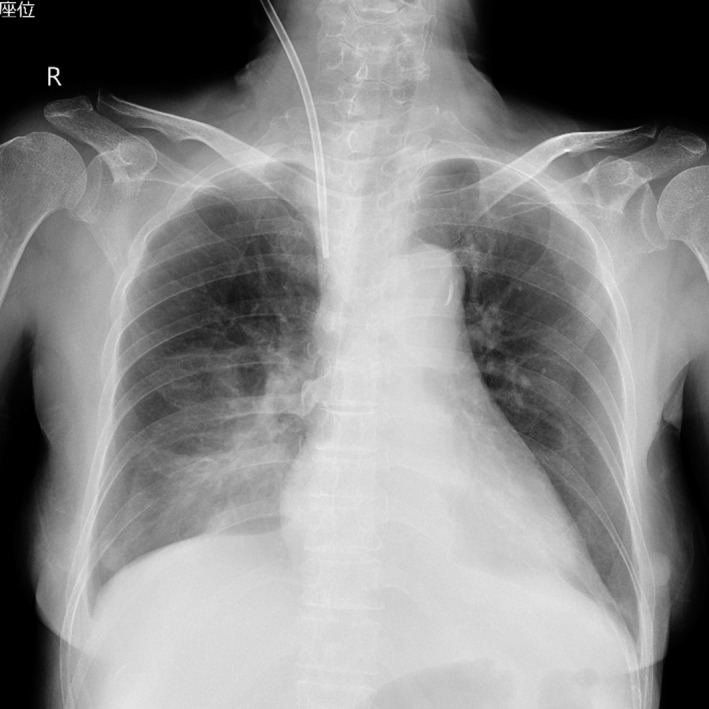
Chest X‐ray after the insertion of a double‐lumen catheter to check the catheter location. This figure shows the catheter positioned appropriately

**Figure 2:**
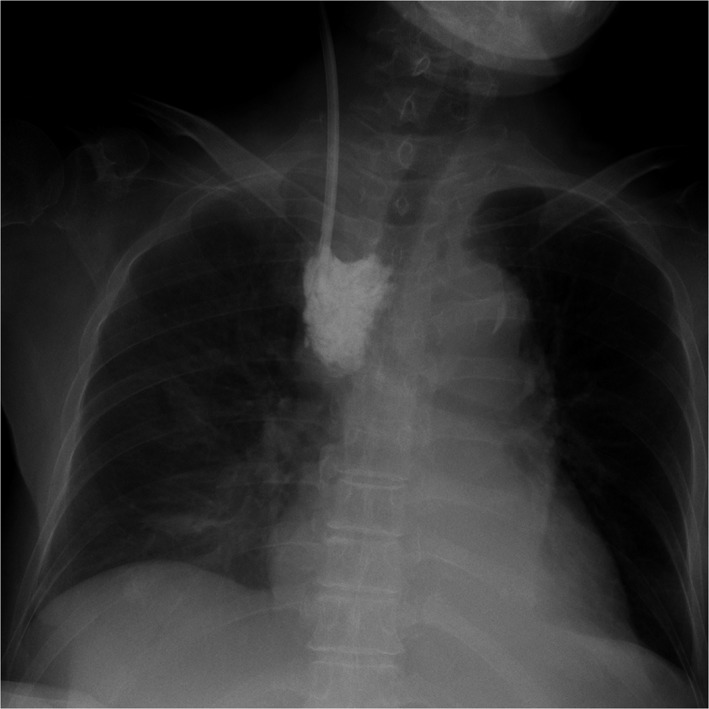
Chest X‐ray after the injection of a contrast agent in the blood return line of the double‐lumen catheter. Contrast agent is visible in the mediastinum

**Figure 3:**
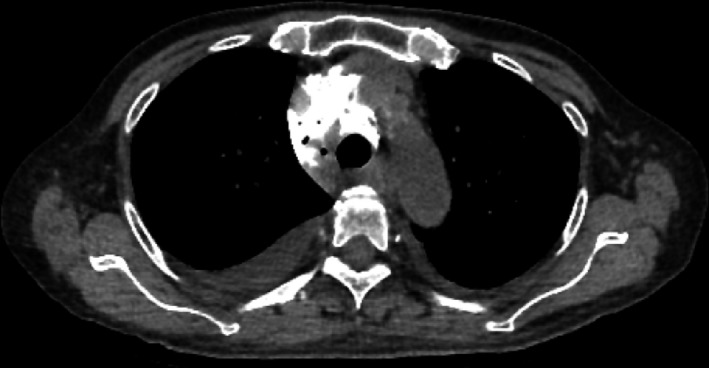
Chest CT after the injection of a contrast agent in the blood return line of the double‐lumen catheter. Contrast agent is visible in the mediastinum

**Figure 4:**
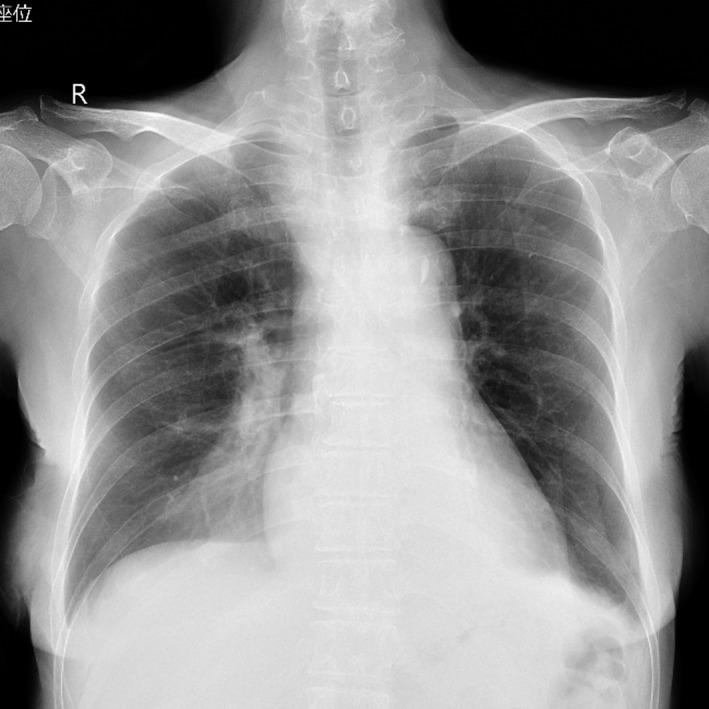
Chest X‐ray obtained 4 days after the contrast agent injection. This figure shows that the contrast agent disappeared

We believe that the catheter passed through the vessel wall diagonally and that the initial blood regurgitation originated from the location indicated by an arrow (Figure 5).

**Figure 5:**
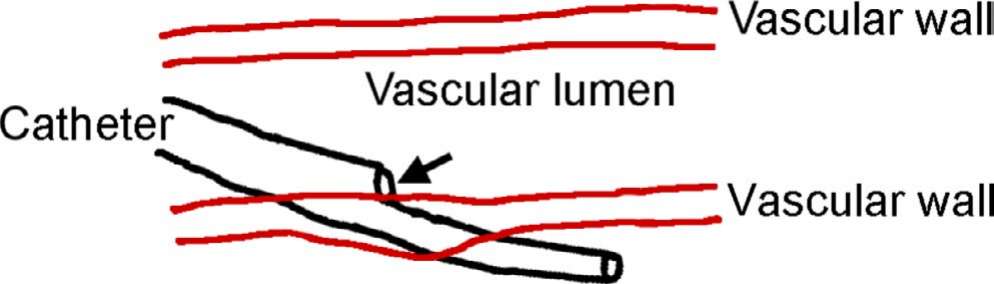
This image shows how the catheter passed through the vessel wall diagonally, explaining the blood regurgitation in the blood removal line. The blood removal line is indicated by an arrow

There have been several reports of a catheter having been inserted into the mediastinum through a central vein.^1^ Checking for blood regurgitation in both the blood removal and return lines is recommended.

## CONFLICTS OF INTEREST

The authors have no conflicts of interest to declare with regard to this report.

## AUTHOR CONTRIBUTIONS

TK: drafted the manuscript. All authors contributed to the therapy and the manuscript preparation and revision. All authors consented to be held accountable for the present report.

## INFORMED CONSENT

We ensured the patient's anonymity and obtained written consent for the present report.
